# BH3 Peptides Induce Mitochondrial Fission and Cell Death Independent of BAX/BAK

**DOI:** 10.1371/journal.pone.0005646

**Published:** 2009-05-21

**Authors:** Emelyn H. Shroff, Colleen M. Snyder, G. R. Scott Budinger, Manu Jain, Teng-Leong Chew, Satya Khuon, Harris Perlman, Navdeep S. Chandel

**Affiliations:** 1 Department of Medicine, Northwestern University Medical School, Chicago, Illinois, United States of America; 2 Cell imaging facility, Northwestern University Medical School, Chicago, Illinois, United States of America; 3 Department of Cell and Molecular Biology Northwestern University Medical School, Chicago, Illinois, United States of America; Roswell Park Cancer Institute, United States of America

## Abstract

BH3 only proteins trigger cell death by interacting with pro- and anti-apoptotic members of the BCL-2 family of proteins. Here we report that BH3 peptides corresponding to the death domain of BH3-only proteins, which bind all the pro-survival BCL-2 family proteins, induce cell death in the absence of BAX and BAK. The BH3 peptides did not cause the release of cytochrome c from isolated mitochondria or from mitochondria in cells. However, the BH3 peptides did cause a decrease in mitochondrial membrane potential but did not induce the opening of the mitochondrial permeability transition pore. Interestingly, the BH3 peptides induced mitochondria to undergo fission in the absence of BAX and BAK. The binding of BCL-X_L_ with dynamin-related protein 1 (DRP1), a GTPase known to regulate mitochondrial fission, increased in the presence of BH3 peptides. These results suggest that pro-survival BCL-2 proteins regulate mitochondrial fission and cell death in the absence of BAX and BAK.

## Introduction

The BCL-2 family of proteins are critical regulators in the process of cell death [1]. Characterized by their conserved BCL-2 Homology (BH) domains, these proteins are divided into groups that have pro-survival and pro-apoptotic functions. The pro-apoptotic group is divided into the single BH3 domain containing proteins and the multi BH3 domains proteins, BAX and BAK [2]. BH3 only proteins activate BAX and BAK to induce the permeabilization of the outer mitochondrial membrane (MOMP), and the subsequent release of cytochrome c [3]. In addition to the physical changes required for the release of cytochrome c, a remarkable alteration during the process of cell death is that the interconnected mitochondria network undergoes morphological changes from long tubular, to short punctiform structures [4]. The short punctated structures reflect fragmentation of the mitochondria, a process referred to as mitochondrial fission.

The mammalian fission machinery to date consists of DRP1, FIS1 and OPA-1. FIS1 is distributed throughout the outer mitochondrial membrane and Dynamin related protein-1 (DRP-1) is localized in the cytosol [5]. DRP1 and FIS1 control the fission of outer mitochondrial membrane while OPA1 regulates fusion of the inner membrane. DRP1 translocate and co-localizes with BAX on the surface of the outer mitochondrial membrane during early stages of apoptosis [6]. DRP1 inhibition delays or partially inhibits cell death suggesting a role for mitochondrial fission in cell death [7], [8]. Furthermore, a small molecule that inhibits DRP-1 prevents BH3-only protein dependent cytochrome c release in isolated mitochondria [9]. In contrast other reports indicate that the downregulaton of hFIS1 or DRP1 prevents mitochondrial fission but not BAX/BAK dependent cell death during apoptosis [10]. The overexpression of hFIS1 does not induce death in cells devoid of BAX and BAK [11]. However, these cells still undergo mitochondrial fragmentation indicating that mitochondrial fission is distinct from BAX/BAK dependent cell death. Recent studies further support that fission and cytochrome c induced cell death are discrete events [12]. Nevertheless, OPA-1 mediated cristae remodeling is required for cytochrome c release [13]. Thus, the role of the fission machinery in cell death is complex [14].

Mitochondrial fission is an evolutionarily conserved process and has been suggested to regulate cell death in *C. elegans* [15]. Interestingly, in this model organism the multi BH3 proteins BAX and BAK are not present, and cytochrome c release does not play a role in initiating cell death [16]. Rather, binding of the BCL-2 like pro-survival protein CED-9, by a BH3-only protein EGL-1, is the key initiation event in the apoptotic pathway, and induces mitochondrial fission [15]. The overexpression of a dominant negative DRP-1 mutant increased survival of cells in this model. However, recent evidence suggests that the role of DRP1 in cell death is distinct from its role in mitochondrial fission in *C. elegans* [17]. Given the fact that BH3 proteins binding to prosurvival BCL-2 proteins can regulate both mitochondrial fission and cell death in *C. elegans*, here we have uncovered that negating the pro-survival proteins by BH3 peptides can also lead to mitochondrial fission and cell death in a mammalian system devoid of BAX and BAK.

## Results

### BH3 peptides induce cell death in the absence of BAX, BAK and BOK

**Figure 1:**
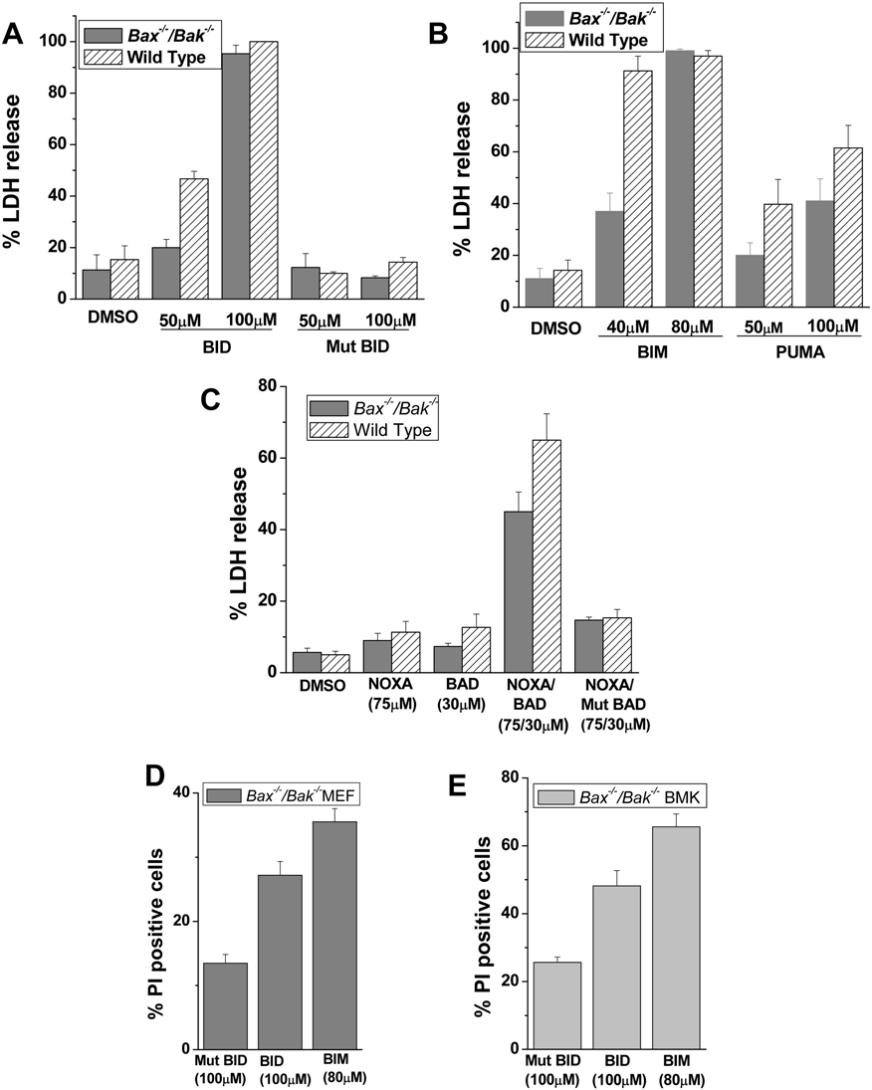
BH3 peptides induce cell death in *Bax^−/−^/Bak^−/−^* MEFs. (A) Percentage of cell death measured by LDH release at 24 hours following treatment of wild type and *Bax^−/−^/Bak^−/−^* MEFs with BID or mutant BID BH3 peptide (50 µM and 100 µM). Mean values±SEMs of 3 independent experiments are shown. (B) Percentage of LDH release from wild type and *Bax^−/−^/Bak^−/−^* MEFs after treatment with BIM (40 µM and 80 µM) or PUMA (50 µM and 100 µM) BH3 peptide for 24 hours. Mean values±SEMs of 3 independent experiments are shown. (C) Wild type and *Bax^−/−^/Bak^−/−^* MEFs were treated with NOXA BH3 peptide (75 µM), BAD BH3 peptide (30 µM), NOXA BH3 peptide (75 µM)+BAD BH3 peptide (30 µM) or NOXA BH3 peptide (75 µM)+mutant BAD BH3 peptide (30 µM). Cell death was measured by LDH released at 24 hours. Mean values±SEMs of 3 independent experiments are shown. (D and E) Percentage of PI positive *Bax^−/−^/Bak^−/−^* MEFs and *Bax^−/−^/Bak^−/−^* Baby Mouse Kidney (BMK) epithelial cells after 1 hour of treatment with BID BH3 peptide (100 µM), mutant BID BH3 peptide (100 µM) or BIM BH3 peptide (80 µM). Mean values±SEMs of 3 independent experiments are shown.

BAX or BAK are the essential regulators for permeabilization of the outer mitochondrial membrane. Previous published studies [3], [18], have demonstrated that several death stimuli including anoxia, growth factor withdrawal, DNA damaging agents fail to induce cell death in the absence of BAX and BAK. These various death stimuli likely activate BH3-only proteins which bind and neutralize a fraction of pro-survival BCL-2 proteins to lower the threshold for the activation of BAX and BAK. To test negation of prosurvival BCL-2 proteins in the absence of BAX and BAK can trigger death, we treated *Bax^−/−^/Bak^−/−^* MEFs with peptides spanning the BH3 domains of the BH3 only proteins. These peptides have been previously demonstrated to have high binding specificity to pro-survival proteins [19]–[21]. These peptides can be administered to cells at concentrations that are likely to bind to a large fraction of pro-survival BCL-2 proteins. The BH3 peptides BID, BIM and PUMA are known to bind all BCL-2 pro-survival proteins. By contrast, NOXA BH3 peptide specifically binds MCL-1 and BAD binds BCL-2, BCL-X_L_, BCL-w and A1. The BH3 peptides are tagged with 8-D arginines which allows for rapid uptake by cells (Figure S1). Treatment with the BID BH3 peptide (100 µM) resulted in similar levels of cell death as measured by LDH release in wild type and *Bax^−/−^/Bak^−/−^* MEFs at 24 hours (Figure 1A). As a control, cells were treated with a mutant BID BH3 peptide in which the conserved residues leucine and aspartate were mutated to alanine. The mutation of these two critical amino acids has been previously shown to disrupt binding to all pro-survival BCL-2 proteins [19], [21]. As predicted, treatment with the mutant BID peptide did not induce cell death in wild type or *Bax^−/−^/Bak^−/−^* MEFs. The overexpression of Bcl-X_L_ slightly prevented BID BH3 induced cell death (Figure S2). BIM or PUMA BH3 peptides also induced cell death in wild type or *Bax^−/−^/Bak^−/−^* MEFs (Figure 1B). Treatment of wild type and *Bax^−/−^/Bak^−/−^* MEFs with NOXA or BAD peptides alone did not induce cell death in either cell lines, whereas the combination of both NOXA and BAD peptides induced cell death in wild type or *Bax^−/−^/Bak^−/−^* MEFs (Figure 1C). To ensure that the cell death was not due to high concentration of the peptides, we designed a mutant version of the BAD peptide, containing two mutations at the conserved residues leucine and aspartate (mutated to alanine). The mutant BAD peptide in combination with NOXA peptide did not cause cell death. To delineate the kinetics of cell death, we treated *Bax^−/−^/Bak^−/−^* MEFs with BH3 peptides for 1 hour and assessed cell death by propidium iodide (PI) staining (Figure 1D). Treatment with BID and BIM BH3 peptides resulted in significant cell death in *Bax^−/−^/Bak^−/−^* MEFs compared to the mutant BID BH3 peptide. In order to rule out that this finding was not restricted to embryonic fibroblasts, we tested for PI staining in an epithelial cell line derived from kidneys of BAX and BAK deficient mouse (referred to as BMK). BMK cells also displayed significant cell death after 1 hour of BID or BIM peptide treatments (Figure 1E).

**Figure 2:**
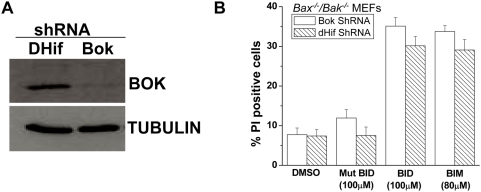
BH3 peptides induce cell death in the absence of BAX, BAK and BOK. (A) Western blot demonstrating levels of BOK protein levels in *Bax^−/−^/Bak^−/−^* MEFs stably transfected with shRNA against BOK. Stable *Bax^−/−^/Bak^−/−^* MEFs expressing a shRNA hairpin against *D. melanogaster* HIF (dHif) served as a control. (B) *Bax^−/−^/Bak^−/−^* MEFs stably transfected with a shRNA against BOK or dHIF were treated with BID BH3 peptide (100 µM), mutant BID BH3 peptide (100 µM) or BIM BH3 peptide (80 µM) and after 1 hour the percentage of PI positive cells was analyzed by FACs analysis. Mean values±SEMs of 3 independent experiments are shown.

The BCL-2 related ovarian killer (BOK) is another pro-apoptotic protein similar to BAX and BAK proteins, in that it contains BH1, BH2 and BH3 domains [22]. In the absence of BAX and BAK we therefore evaluated whether BOK was required for BH3 peptide induced cell death. PCR and western blot analysis both revealed that BOK mRNA and protein were expressed in the *Bax^−/−^/Bak^−/−^* embryonic fibroblast cells and in epithelial kidney cells (data not shown). We retrovirally infected *Bax^−/−^/Bak^−/−^* MEFs with short hairpin RNA against BOK, or a control shRNA containing drosophilia HIF (DHIF) (Figure 2A). BID and BIM BH3 peptides induced death in cells containing shRNA against BOK or DHIF (Figure 2B). Collectively these data demonstrate that BH3 peptides that can bind to all the pro-survival BCL-2 proteins can induce cell death independent of BAX, BAK and BOK.

### Caspase inhibition does not inhibit BH3 peptides induced cell death

**Figure 3:**
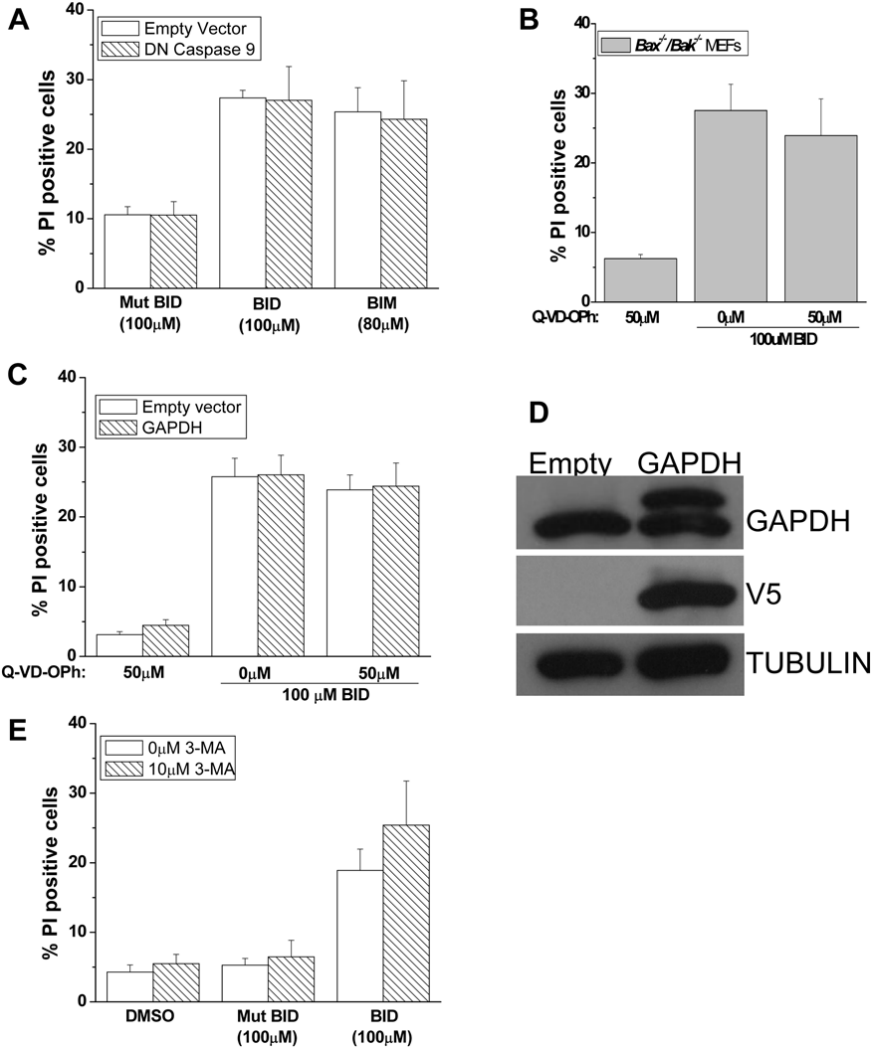
Caspase inhibition does not inhibit BH3 Peptides induced cell death. (A) *Bax^−/−^/Bak^−/−^* MEFs stably expressing a dominant negative caspase 9, or empty vector were treated with indicated peptides and after 1 hour the percentage of PI positive cells was analyzed by FACs analysis. Mean values±SEMs of 3 independent experiments are shown. (B) *Bax^−/−^/Bak^−/−^* MEFs were treated with Q-VD-OPh (50 µM, broad range caspase inhibitor) for 1 hour prior to adding BID peptide (100 µM) for 1 hour. Percentage cell death was determined by assessing for cell staining positive for propidium iodine by flow cytometry. Mean values±SEMs of 3 independent experiments are shown. (C) *Bax^−/−^/Bak^−/−^* MEFs expressing GAPDH, or control empty vector were treated with indicated peptides in the presence or absence of Q-VD-OPh (50 µM) for 1 hour. Percentage of PI positive cells was analyzed by flow cytometry. (D) Western blot demonstrating levels of GAPDH protein over-expression in *Bax^−/−^/Bak^−/−^* MEFs. GAPDH was tagged to V5, therefore both endogenous and overexpressed levels could be identified. Empty vector was used as a transfection control. Bottom panel show tubulin levels, a loading control. (E) *Bax^−/−^/Bak^−/−^* MEFs were treated with 3-Methyladenine (10 µM, autophagy inhibitor) for 1 hour prior to adding BID BH3 peptide (100 µM) or mutant BID BH3 peptide (100 µM) for 1 hour. Percentage cell death was determined by assessing for cell staining positive for propidium iodine by flow cytometry. Mean values±SEMs of 3 independent experiments are shown.

Caspases are key effectors of apoptosis. We evaluated whether the peptide induced cell death was caspase dependent. To assess whether caspase 9 was involved, we inhibited endogenous caspase-9 of *Bax^−/−^/Bak^−/−^* MEFs by the overexpressing dominant negative caspase-9 [23]. BID and BIM peptides still induced cell death in the presence of the dominant negative caspase-9 (Figure 3A). To test whether pan caspase inhibition would prevent cell death, *Bax^−/−^/Bak^−/−^* MEFs were treated with the pan-caspase inhibitor quinoline-Val-Asp(ome)-CH2-O-phenoxy (Q-VD-OPh). BH3 peptides induce cell death in the presence of Q-VD-OPh (Figure 3B). These results indicate that BH3 peptides might be activating caspase independent cell death in the *Bax^−/−^/Bak^−/−^* MEFs. Caspase-independent cell death (CICD) can be prevented by the overexpression of the glycolytic enzyme glyceraldehyde-3-phosphate dehydrogenase (GAPDH) in the presence of a pan caspase inhibitor [24]. However, BH3 peptides still induced cell death in *Bax^−/−^/Bak^−/−^* MEFs overexpressing GAPDH in the presence of the broad spectrum caspase inhibitor Q-VD-OPh (Figure 3C and 3D). We also tested whether autophagy contributed to the BH3 peptide induced cell death. Autophagy induced cell death can occur in the absence of BAX and BAK [25]. To test whether *Bax^−/−^/Bak^−/−^* MEFs died by activating autophagy, we utilized a well characterized inhibitor of autophagy, 3-methyladenine (3-MA). *Bax^−/−^/Bak^−/−^* MEFs were pretreated with 3-MA followed by BID BH3 peptide in the presence of 3-MA. 3-MA did not protect against BID BH3 peptide cell death (Figure 3E), therefore suggesting that the BH3 peptide induced cell death in the absence of BAX and BAK was not mediated by autophagy. Collectively, these experiments demonstrate that caspases, caspase independent cell death regulated by GAPDH or autophagy are not likely contributing to the cell death.

### BH3 peptides do not cause the release cytochrome c in *Bax^−/−^/Bak^−/−^* MEFs

**Figure 4:**
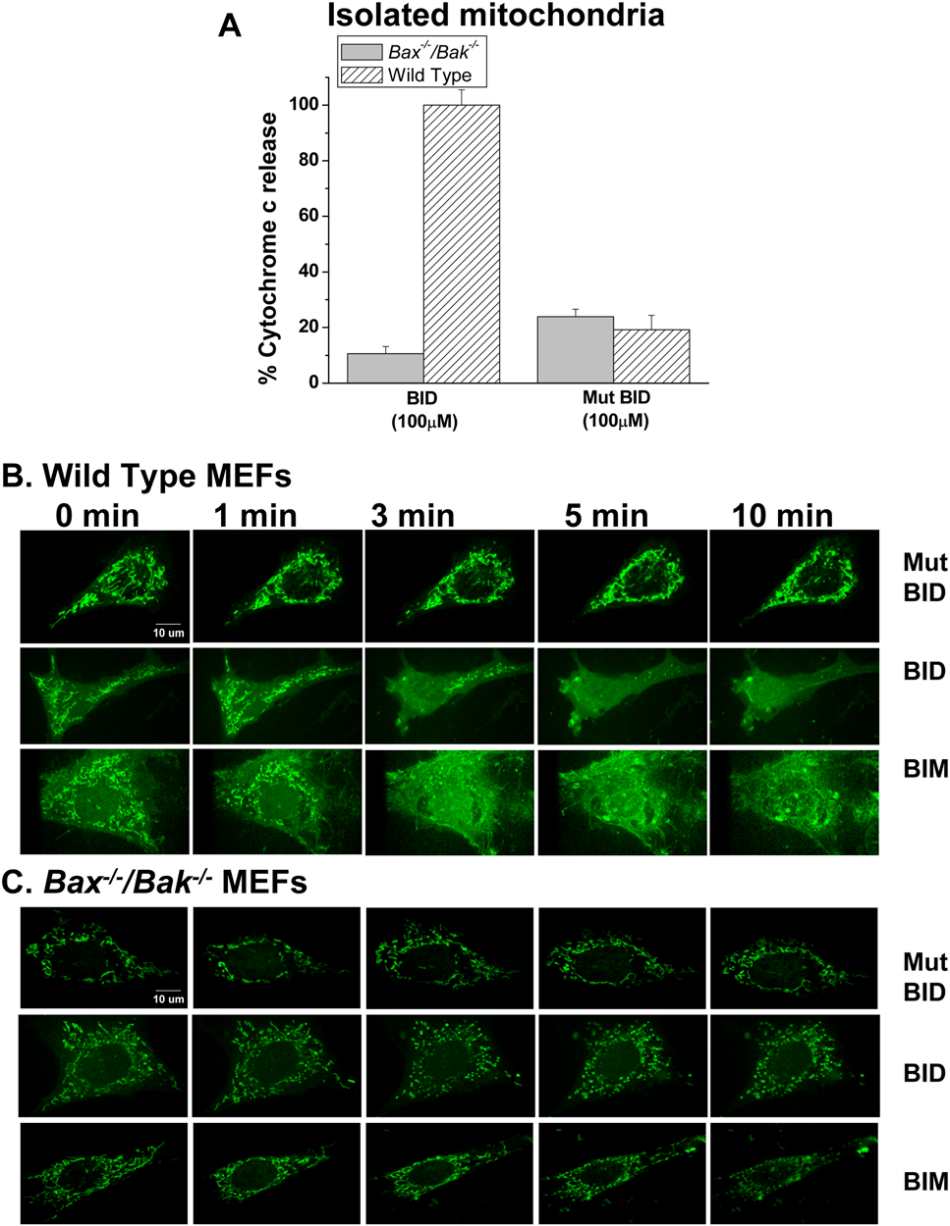
BH3 Peptides do not induce release of cytochrome c from *Bax^−/−^/Bak^−/−^* isolated mitochondria or MEFs. (A) Isolated mitochondria of wild type and *Bax^−/−^/Bak^−/−^* MEFs were treated with BID BH3 peptide (100 µM) or mutant BID BH3 peptide (100 µM). Cytochrome c released from isolated mitochondria was determined by ELISA read at 450 nm with wavelength correction at 540 nm. Mean values±SEMs of 3 independent experiments are shown. (B and C) Wild type MEFs and *Bax^−/−^/Bak^−/−^* MEFs respectively were stably transfected with cytochrome c tagged with GFP and time lapse microscopy was performed to assess for cytochrome c release upon treatment with BID BH3 peptide (100 µM), mutant BID BH3 peptide (100 µM) or BIM BH3 peptide (80 µM) Pictures were taken prior to peptide treatment and at 1 minute intervals post peptide treatment.

To evaluate whether the BID BH3 peptide induces cytochrome c release, the BID BH3 peptide was added to isolated mitochondria from wild type and *Bax^−/−^/Bak^−/−^* MEFs. BID BH3 peptide induced cytochrome c release in the wild type MEFs but not in the *Bax^−/−^/Bak^−/−^* MEFs. The mutant BID BH3 peptide did not cause cytochrome c release in either wild type or *Bax^−/−^/Bak^−/−^* isolated mitochondria (Figure 4A). Because cytochrome c was not released from the isolated mitochondria of *Bax^−/−^/Bak^−/−^* MEFs, we further evaluated whether cytochrome c was released in live cells. Wild type and *Bax^−/−^/Bak^−/−^* MEFs were transfected with cytochrome c tagged with GFP and time lapse microscopy was performed. While the mutant BID peptide did not display release of cytochrome c from the mitochondria, both BID and BIM peptides induced cytochrome c release in the wild type cells as demonstrated by the diffusion pattern of the GFP throughout the cell (Figure 4B). However, *Bax^−/−^/Bak^−/−^* MEFs treated with BID and BIM peptides, did not display diffused GFP within the cell, which is indicative of a lack of cytochrome c release (Figure 4C).

**Figure 5:**
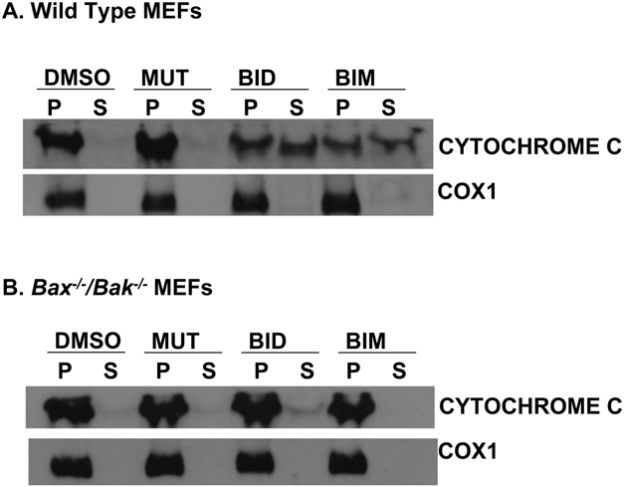
BH3 Peptides do not induce release of cytochrome c in *Bax^−/−^/Bak^−/−^* MEFs. (A and B) Wild type and *Bax^−/−^/Bak^−/−^* MEFs were treated with DMSO, Mutant BID BH3 peptide (100 µM), BID BH3 peptide (100 µM) or BIM BH3 peptide (100 µM) for 1 hour. Cytochrome c localization was assessed in the mitochondrial and cytosolic fractions by Western Blot analysis. Cytochrome c oxidase subunit 1 (COX-1) antibody was used as a control to ensure proper fractionation and loading of mitochondrial pellet.

Western blot analysis further confirmed that the mutant BID, BID or BIM BH3 peptides did not induce the release of cytochrome c in *Bax^−/−^/Bak^−/−^* MEFs as detected by the retention of cytochrome c in the mitochondrial pellet fraction (Figure 5B). On the contrary, treatment of wild type MEFs with BID or BIM BH3 peptides but not the mutant BID BH3 peptide induced the release of cytochrome c into the cytosol as detected by the presence of cytochrome c in the supernatant fraction (Figure 5A). This data further indicate that *Bax^−/−^/Bak^−/−^* MEFs cell death is not through the release of cytochrome c.

### BH3 peptides induce a decrease in mitochondrial membrane potential in *Bax^−/−^/Bak^−/−^* MEFs independent of the permeability transition pore

**Figure 6:**
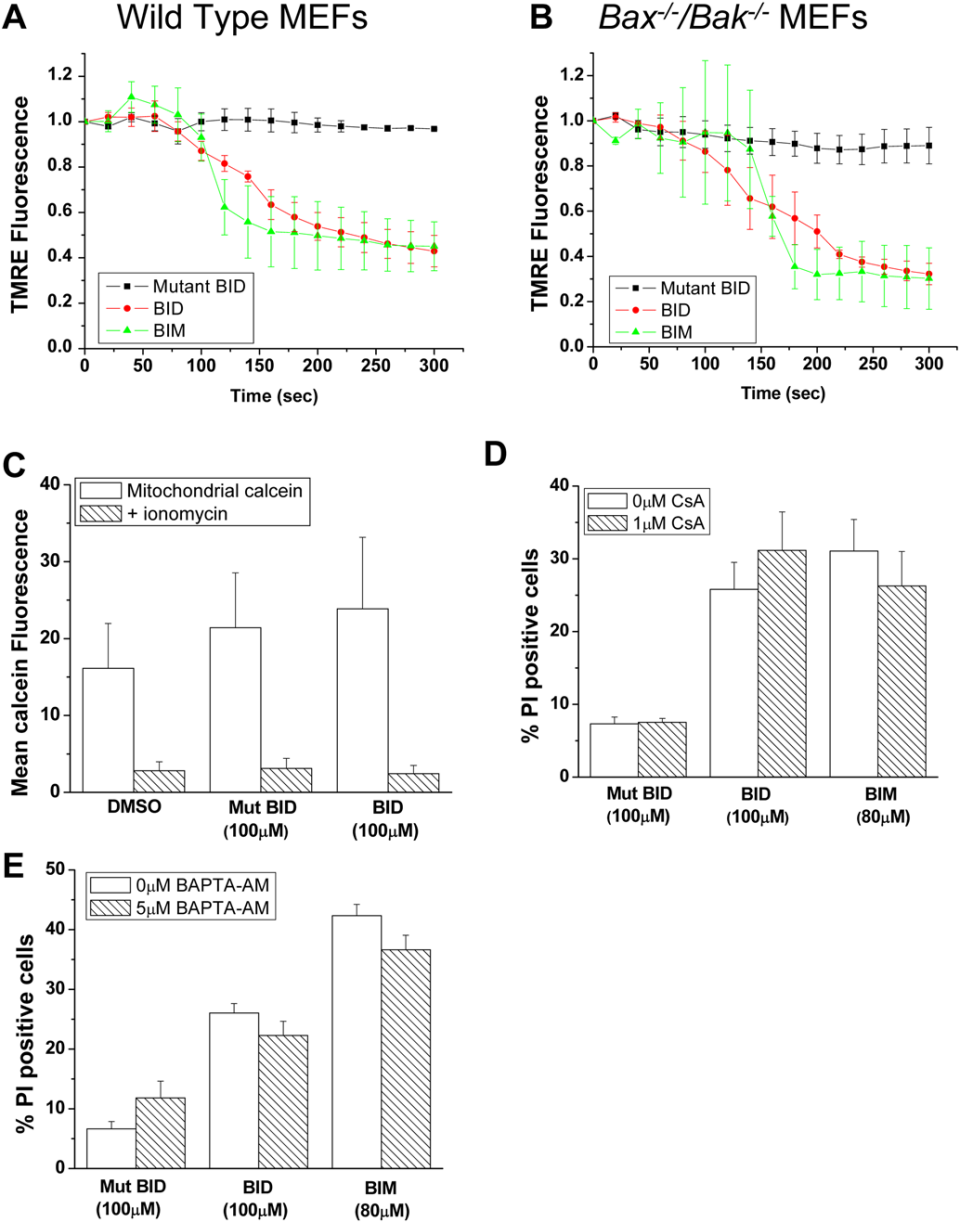
BH3 Peptides induces a decrease in mitochondrial membrane potential but does not engage the Permeability Transition Pore. (A and B) Quantification of TMRE release from wild type and *Bax^−/−^/Bak^−/−^* MEFs respectively, as an output of mitochondrial membrane depolarization. Mitochondria were loaded with 50 nM TMRE and treated BID BH3 peptide (100 µM), mutant BID BH3 peptide (100 µM) or BIM BH3 peptide (80 µM). Time lapse imaging was taken at 20 second intervals. Mean values±SEMs of 3 independent experiments are shown. (C) *Bax^−/−^/Bak^−/−^* MEFs treated for 1 hour with DMSO, BID BH3 peptide (100 µM), mutant BID BH3 peptide (100 µM), followed by 100 nM of calcein-AM labeling (and quenched with 0.4 mM Cobalt Chloride) in the presence or absence of ionomycin (500 nM). Mean calcein fluorescence was measured at 530 nm by flow cytometry. Results are expressed as mean values±SEMs of 5 independent experiments. (D) *Bax^−/−^/Bak^−/−^* MEFs were treated with cyclosporine A (1 µM, CsA) and treated with BID BH3 peptide (100 µM), mutant BID BH3 peptide (100 µM) or BIM BH3 peptide (80 µM) for 1 hour. Percentage cell death was determined by assessing for PI staining by flow cytometry. Mean values±SEMs of 3 independent experiments are shown. (E) *Bax^−/−^/Bak^−/−^* MEFs were treated with BAPTA-AM (5 µM) for 15 minutes prior to adding BID BH3 peptide (100 µM), mutant BID BH3 peptide (100 µM) or BIM BH3 peptide (80 µM) for 1 hour. Percentage cell death was determined by assessing for cell staining positive for propidium iodine by flow cytometry. Mean values±SEMs of 3 independent experiments are shown.

Typically, cells undergo a loss of mitochondrial membrane potential upon cytochrome c release [26]. To assess mitochondrial membrane potential in wild-type or *Bax^−/−^/Bak^−/−^* MEFs, we loaded cells with fluorescent dye tetramethylrhodamine ester (TMRE). BIM and BID BH3 peptide induced a decrease in TMRE fluorescence compared to the mutant BID BH3 peptide in both wild-type or *Bax^−/−^/Bak^−/−^* MEFs indicative of mitochondrial membrane depolarization (Figure 6A and B).

To test whether the permeability transition pore (PTP) is responsible for the loss in mitochondrial membrane potential, we utilized the release of calcein from the mitochondria. *Bax^−/−^/Bak^−/−^* MEFs were loaded with calcein and cobalt chloride, which quenches calcein signal in the cytosol. Thus the remaining signal is calcein retained in the mitochondria. Calcein is released upon PTP opening with agents such as ionomycin. BID BH3 peptide treated *Bax^−/−^/Bak^−/−^* MEFs had comparable mitochondrial calcein retention to that of DMSO and mutant BID treated *Bax^−/−^/Bak^−/−^* MEFs, which could be decreased to baseline upon the addition of ionomycin (Figure 6C). We further assessed for the involvement of the PTP in the peptide induced cell death by its direct inhibition using 1 µM of cyclosporine A (CsA). Inhibiting the PTP with CsA did not protect *Bax^−/−^/Bak^−/−^* MEFs from peptide induced cell death (Figure 6D). We confirmed that 1 µM CsA was efficient at inhibiting the permeability transition pore by assessing for calcein retention in the mitochondria following treatment with ionomycin (Figure S3). Calcium is one of the major regulators of PTP opening. We tested whether calcium was involved in the BH3 peptide induced cell death in the *Bax^−/−^/Bak^−/−^* MEFs. The calcium chelator BAPTA-AM did not prevent BID or BIM BH3 peptide induced cell death (Figure 6E). These data suggest that BH3 peptides induced mitochondrial membrane depolarization without invoking PTP opening.

### BH3 peptides induce mitochondrial fragmentation in *Bax^−/−^/Bak^−/−^* MEFs

**Figure 7:**
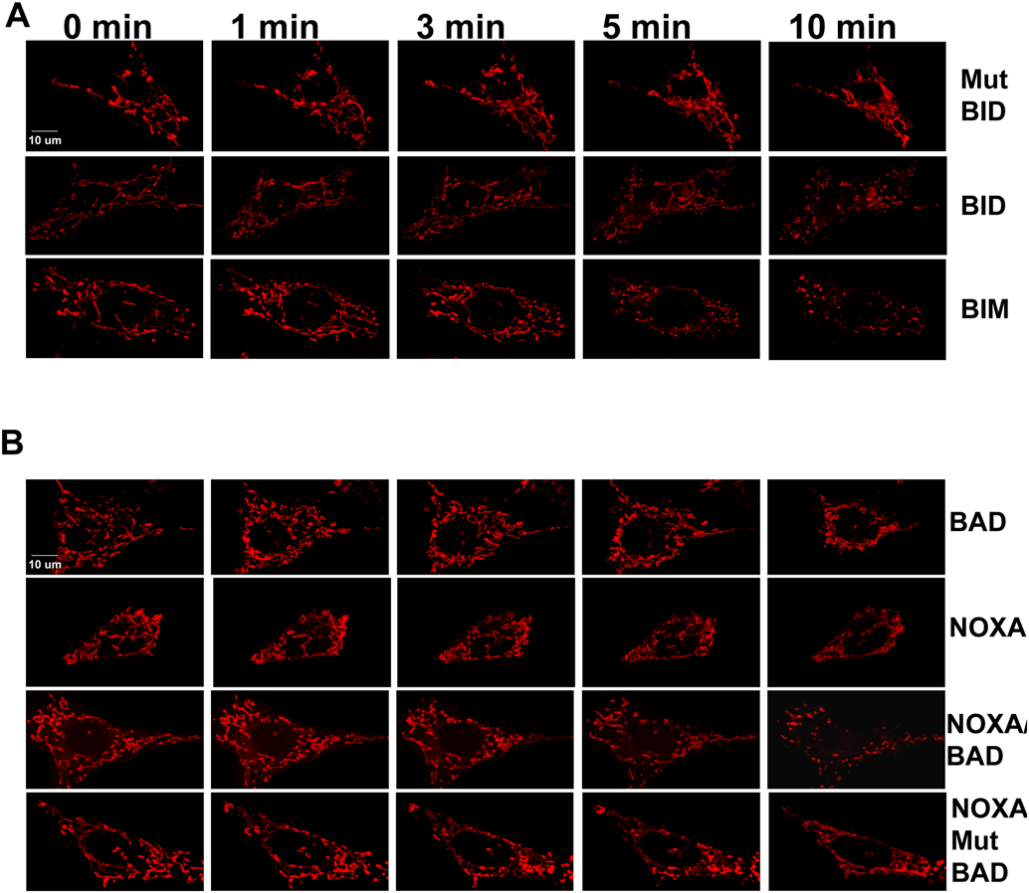
BH3 peptides induce mitochondrial fragmentation of *Bax^−/−^/Bak^−/−^* MEFs. (A) Mitochondria of *Bax^−/−^/Bak^−/−^* MEFs were stained with Mitotracker Red CMXRos (50 nM) and time lapse microcopy was performed. Pictures were taken prior to and at 1 minute intervals following treatment with 100 µM of mutant BID, BID and BIM BH3 peptides. (B) Time lapse microscopy of *Bax^−/−^/Bak^−/−^* MEFs stained with Mitotracker RedCMXRos (50 nM). Cells were treated with sensitizer peptides, NOXA (75 µM) or BAD BH3 peptide (30 µM) alone, or in combination. The combination of NOXA (75 µM) and mutant BAD BH3 peptide (30 µM) was also analyzed.

We observed a change in mitochondrial morphology from long tubular to a small punctuated pattern of cytochrome c-GFP in *Bax^−/−^/Bak^−/−^* MEFs (Figure 4C). To further confirm these morphological changes in mitochondria, *Bax^−/−^/Bak^−/−^* MEFs were stained with a fluorescent dye, Mitotracker CMX-ROS. *Bax^−/−^/Bak^−/−^* MEFs treated with mutant BID BH3 peptide displayed normal mitochondrial dynamics, and mitochondria appeared tubular and elongated (Figure 7A top panel). By contrast, *Bax^−/−^/Bak^−/−^* MEFs treated with the BID and BIM BH3 peptides displayed short, spherical mitochondria (Figure 7A middle and bottom panel respectively), indicative of mitochondrial fragmentation. *Bax^−/−^/Bak^−/−^* MEFs treated with BAD or NOXA BH3 peptides, did not reveal any changes in the mitochondrial morphology and maintained the elongated tubular structures (Figure 7B). However, the combination of BAD and NOXA peptides induced a change in the mitochondrial morphology from tubular and elongated structures to smaller spherical structures (Figure 7B) similar to those observed with BID and BIM. As a control, the combination of NOXA with a mutant BAD peptide did not induce changes in mitochondrial structure (Figure 7B, bottom panel). These data demonstrate that mitochondria underwent fragmentation only when the pro-survival proteins were neutralized with BH3 peptides.

**Figure 8:**
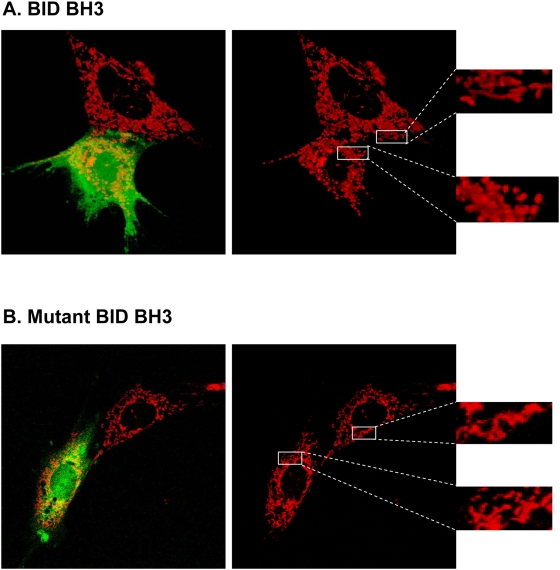
Micro-injection of BH3 peptides induces mitochondrial fragmentation of *Bax^−/−^/Bak^−/−^* MEFs. (A and B) Mitochondria of *Bax^−/−^/Bak^−/−^* MEFs were stained with Mitotracker Red CMXRos (50 nM) followed by microinjection of 100 µM BID (A) or mutant BID peptide (B) that does not contain 8D-arginines. Injected cells were tracked by dextran FITC.

To further confirm that the change in mitochondrial morphology was not dependent on peptide uptake by the cell, we performed microinjection of BID BH3 and mutant BID BH3 peptides that did not contain the 8-D arginine sequence in combination with FITC dextran as a marker for injected cells. We captured images of cells that were injected next to non injected cells to show the difference in mitochondrial morphology. As shown in figure 8A when *Bax^−/−^/Bak^−/−^* MEF were microinjected with the BID BH3 peptide, the mitochondrial morphology changed from long tubular structures to small spherical structures, while the mutant BID BH3 peptide did not induce a change in mitochondrial structure (Figure 8B) upon microinjection.

### BH3 peptides induce a decrease in mitochondrial aspect ratio of *Bax^−/−^/Bak^−/−^* MEFs

**Figure 9:**
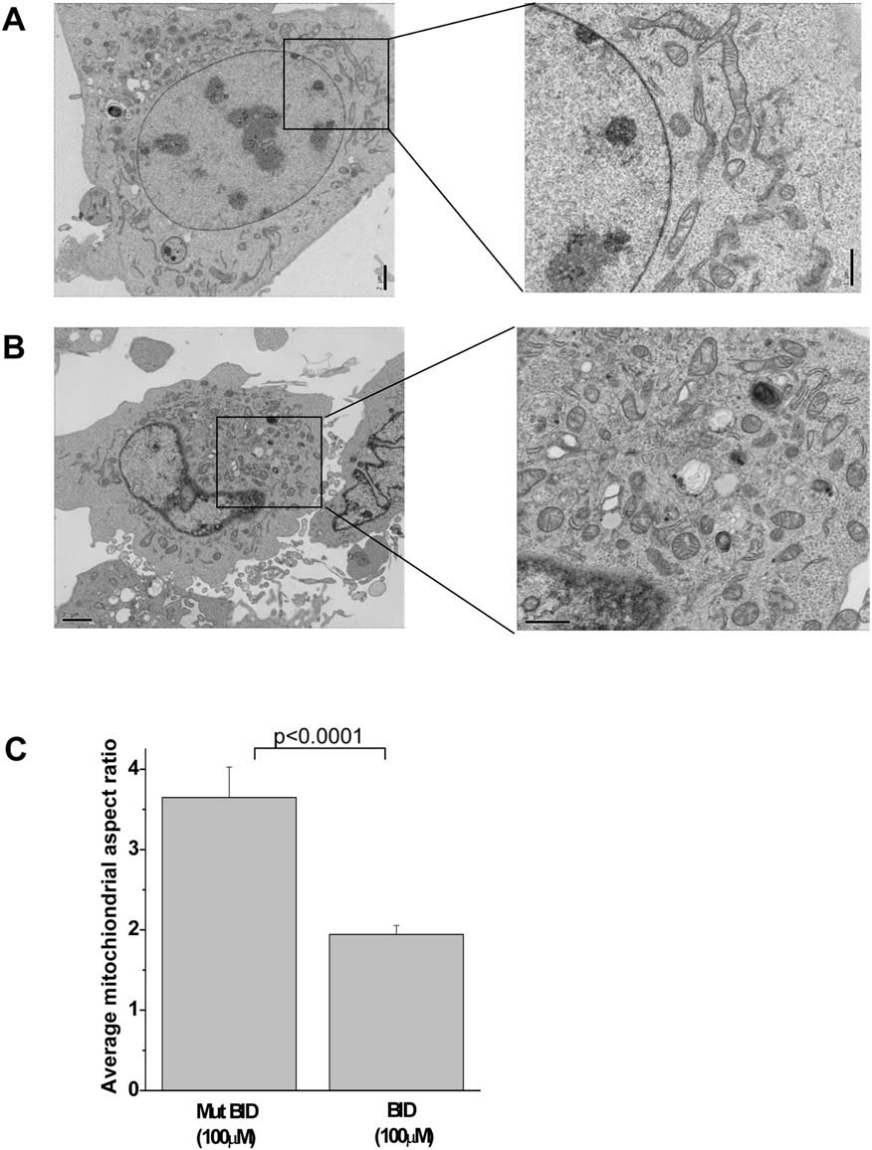
BH3 peptides induce a decrease in mitochondrial aspect ratio of *Bax^−/−^/Bak^−/−^* MEFs. (A) Electron micrograph at 2000× of *Bax^−/−^/Bak^−/−^* MEFs treated with mutant BID BH3 peptide (100 µM). 6000× magnification of indicated area. (B) Electron micrograph at 2000× of *Bax^−/−^/Bak^−/−^* MEFs treated with BID BH3 peptide (100 µM). Indicated regions are at 6000× magnification. (C) Mitochondrial length was determined by analyzing the aspect ratio (AR), (length of major axes/ minor axes). BID treatment resulted in a decrease in AR ratio. P value = 0.0001. Student's unpaired t test was used to assess for statistical significance.

We further verified the change in mitochondrial morphology using electron microscopy. Electron micrographs revealed that *Bax^−/−^/Bak^−/−^* MEFs treated with the mutant BID BH3 peptide contained more elongated mitochondria (Figure 9A) compared to cells treated with BID BH3 peptide, whereby the mitochondria were smaller and appeared more spherical in structures (Figure 9B). Quantitative analysis was performed by measuring the aspect ratio (major axes divide by the minor axes) of the mitochondria. Statistical analysis showed that *Bax^−/−^/Bak^−/−^* MEFs treated with BID BH3 peptide had a reduced aspect ratio compared to the mutant BID BH3 peptide (Figure 9C). These results further confirm that the mitochondria were indeed undergoing fragmentation, resembling that of mitochondrial fission.

### BH3 peptides induce an increase in BCL-X_L_ binding to dynamin-related protein 1 (DRP1)

**Figure 10:**
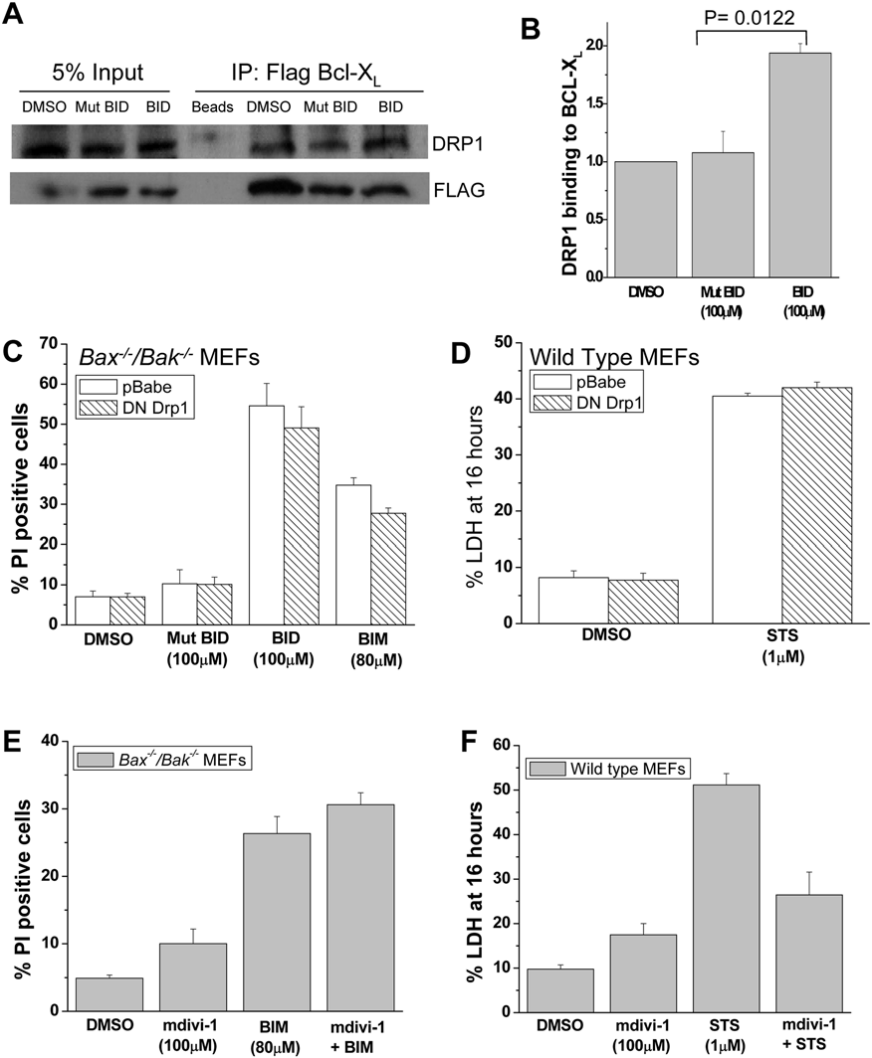
BH3 peptides induce an increase in Bcl-X_L_ binding to dynamin-related protein 1 (DRP1). (A) Immunoblot of coimmunoprecipitation of BCL-X_L_ and DRP1. *Bax^−/−^/Bak^−/−^* MEFs overexpressing Flag BCL-X_L_ were treated with DMSO, BID BH3 peptide (100 µM) or mutant BID BH3 peptide (100 µM). Flag antibody was used for immunoprecipitation. (B) Relative amounts of DRP1 binding to BCL-X_L_ following treatment of mutant BID BH3 peptide (100 µM) or BID BH3 peptides (100 µM). BID BH3 peptide induced a two fold increase in DRP1 binding to BCL-X_L_. P value = 0.0122. Student's unpaired t test was used to assess for statistical significance. (C) *Bax^−/−^/Bak^−/−^* MEFs overexpressing DN DRP1 or empty pBabe construct as a control were treated for 1 hour with DMSO or indicated peptides. Percentage of cell death was determined by LDH release. Mean values±SEMs of 3 independent experiments are shown. (D) Wild Type MEFs overexpressing DN DRP1 or empty pBabe construct as a control were treated for 16 hours with DMSO or staurosporine (1 µM STS). Percentage of cell death was determined by assessing for PI positive cells by flow cytometry. Mean values±SEMs of 3 independent experiments are shown. (E) *Bax^−/−^/Bak^−/−^* MEFs were pretreated with the mitochondrial fission inhibitor mdivi-1 (100 µM) for 30 minutes followed by 1 hour treatment with of BIM BH3 peptide (80 µM). Percentage of cell death was assessed by PI staining. Mean values±SEMs of 3 independent experiments are shown. (F) Wild type MEFs were pretreated with mdivi-1 (100 µM) for 30 minutes followed by 16 hour treatment with staurosporine (1 µM STS). Percentage of cell death was assessed by LDH release. Mean values±SEMs of 3 independent experiments are shown.

Previous published findings showed that the pro-survival protein BCL-X_L_ interacts with the fission machinery protein DRP1 and that this interaction increases the GTPase activity of DRP1 [27]. We therefore investigated whether BCL-X_L_ and DRP1 interact upon treatment with BH3 peptides in *Bax^−/−^/Bak^−/−^* MEFs. *Bax^−/−^/Bak^−/−^* MEFs overexpressing FLAG-tagged BCL-X_L_ were treated with peptides, followed by immunoprecipiation with a FLAG antibody and immunoblotted for DRP1 (Figure 10A). Treatment with the BID BH3 peptide caused a twofold increase in the interaction between DRP-1 and BCL-X_L_ compared to cells treated with the mutant BID BH3 peptide (Figure 10B). To test whether DRP1 is required for the BH3 peptide induced cell death, *Bax^−/−^/Bak^−/−^* MEFs were infected with a retrovirus containing a dominant negative DRP1 (K38A). BIM or BID BH3 peptide induced cell death in *Bax^−/−^/Bak^−/−^* MEFs expressing the dominant negative DRP1 (Figure 10C). The dominant negative DRP1 also failed to prevent staurosporine induced cell death in wild-type MEFs (Figure 10D). Dominant negative DRP-1 also did not prevent mitochondrial fission upon treatment with BH3 peptides (data not shown). Furthermore, the small molecule inhibitor of DRP1, mdivi-1, also did not inhibit cell death in the presence of BIM BH3 peptides (Figure 10E). However, Mdivi-1 did partially inhibit staurosporine induced cell death in wild-type MEFs (Figure 10F). These results suggest that BH3 peptides bind to pro-survival BCL-2 proteins to engage the DRP-1 dependent fission machinery in the absence of BAX and BAK. However, the DRP1 induced fission is not required for BH3 peptide induced cell death. There might be other yet unidentified regulators of fission machinery that might participate in the BH3 peptide induction of cell death.

## Discussion

The BCL-2 protein BAX and BAK induce the mitochondrial outer membrane permeabilization (MOMP) for the release of cytochrome c. Current models indicate that the BH3 only proteins, a subset of the BCL-2 family proteins, are initiators of BAX/BAK dependent MOMP. How BAX and BAK are activated to induce MOMP remains controversial. One model states that a subset of BH3-only proteins bind pro-survival BCL-2 proteins to release other BH3 only proteins that can directly activate BAX/BAK [21], [28]. By contrast, another model states that binding of all pro-survival BCL-2 family proteins by BH3 only proteins is sufficient to activate BAX/BAK [20], [29]. A common feature of the two models is that negation of pro-survival BCL-2 family members is required for BAX/BAK to induce MOMP. Here we demonstrate that cell death can be induced in the absence of the multi BH3 proteins BAX and BAK, upon treatment with peptides corresponding to the BH3 domain of BH3 only pro-apoptotic proteins. Previous studies have characterized that the BH3 peptides indeed bind to pro-survival proteins with strong affinities and specificities. Our data indicates that the BH3 peptides can induce cell death in the combined absence of BAX and BAK through binding of all the pro-survival proteins.

Our observation is reminiscent of the death process observed in *C. elegans*, whereby the neutralization of the pro-survival protein CED-9 by the BH3 only protein EGL-1 can induce both mitochondrial fission and cell death [15]. *C. elegans* do not contain BAX or BAK. A recent report indicates that mitochondrial fragmentation and cell death are distinct events in *C. elegans* [17]. In mammalian cells where BAX and BAK are present, the neutralization of pro-survival proteins by BH3 only proteins triggers rapid activation of BAK and BAK to induce MOMP. However, in mammalian cells deficient for BAX and BAK, we can unmask the evolutionarily conserved mechanism of cell death, in which BH3 only proteins bind pro-survival BCL-2 proteins to initiate both mitochondrial fission and cell death in the absence of BAX and BAK.

Although our findings seem to be in contradiction with previous data that indicate that *Bax^−/−^/Bak^−/−^* MEFs do not undergo cell death upon overexpression of BH3 only proteins, the difference is that the BH3 peptides might bind to a larger pool of pro-survival BCL-2 family proteins. Alternatively, the BH3 peptides can perhaps access pro-survival proteins better than endogenous BH3 proteins. The BH3 peptides have been extensively utilized previously to probe both specificities of BH3 proteins binding to prosurvival BCL-2 proteins and the mechanism by which BH3 proteins induce release of cytochrome c [19]–[21], [28]. Previous data indicate that expression of BH3 only protein BIM which can bind all the pro-survival BCL-2 family proteins can induce cell death in a BAX/BAK dependent manner. By contrast, the expression of the BH3 only protein BAD which only binds a subset of pro-survival BCL-2 family proteins (BCL-2, BCL-X_L_, A1, BCL-w) does not induce cell death [30], [31]. In accordance with these previous studies we also observe that expression of BIM protein and not BAD protein induces BAX/BAK dependent cell death (data no shown). In contrast, treating the cells with BIM BH3 peptide induces cell death even in the absence of BAX and BAK. Other BH3 peptides that can bind all the pro-survival proteins such as BID and PUMA can also induce cell death even in the absence of BAX and BAK. Additionally, neither the NOXA BH3 peptide, which only binds the pro-survival protein MCL-1, nor the BAD BH3 peptide, which binds only BCL-2, BCL-X_L_, A1 and BCL-w, failed to induce cell death. However, the combination of NOXA and BAD BH3 peptides did induce cell death in the absence of BAX/BAK. Furthermore, as controls throughout our study, we utilized when appropriate, either a mutant BID BH3 peptide or mutant BAD BH3 peptides. These peptides contain two amino acid mutated to alanine thus disrupting their binding to pro-survival BCL-2 family proteins [19], [21]. The mutation still allowed for efficient transport of the peptides in cells. These controls indicate that the BH3 peptide killing of *Bax^−/−^/Bak^−/−^* cells is not due non-specific effects such as the 8 D-arginine residues utilized to transport the peptides across cell membranes. Also, these observations in MEFs were corroborated utilizing a BAX/BAK deficient epithelial cell line.

The BH3 peptides did not result in any detectable cytochrome c release from mitochondria isolated from *Bax^−/−^/Bak^−/−^* cells or from intact *Bax^−/−^/Bak^−/−^* cells. These peptides simply do not permeabilize membranes or else they would have released cytochrome c even in the absence of BAX/BAK. These results are consistent with previous findings that BH3 proteins or peptides require BAX/BAK for cytochrome c release [3], [21]. However, the BH3 peptides resulted in depolarization of the mitochondrial membrane potential in the absence of BAX and BAK. Loss of mitochondrial membrane potential can be an initiating event for induction of cell death [32]. Previous studies have indicated that mitochondrial fission can result in loss of mitochondrial membrane potential [7]. Depolarized mitochondria as a result of excessive fission have lower probability to refuse, which therefore leads to an accumulation of fragmented dysfunctional mitochondria [33]. Indeed, we observed that following BH3 peptides treatment, the mitochondrial of *Bax^−/−^/Bak^−/−^* MEFs undergo fission as assessed by staining mitochondria with a fluorescent dye or electron microscopy. Thus, we propose that BH3 peptides induce mitochondrial fission resulting in mitochondrial membrane depolarization in the absence of BAX/BAK.

The role of mitochondrial fission during the process of cell death still remains unclear [14]. An existing idea is that fragmentation of the mitochondria is important for the release of apoptogenic factors such as cytochrome c to induce cell death [7], [8], [34]. This is further supported by the observation that OPA-1 mediated cristae remodeling is required for efficient cytochrome c release. Our data indicate that the mitochondria can undergo fission without the release of cytochrome c, indicating that the two processes are discrete events. This observation is consistent with a recent study by Sheridan et al demonstrating that BH3 proteins can induce mitochondrial fragmentation without cytochrome c release in the presence of BAX/BAK inhibition by a pro-survival protein [12].

The mechanism responsible for the activation of mitochondrial fission and its regulation is still not fully understood [4]. However, it is appreciated that the activation of the fission protein DRP1 is increased under apoptotic conditions [6], [7]. The fact that BAX and BAK deficient cells can still undergo fission [11] suggests that the BAX and BAK are dispensable for initiating mitochondrial fission. By contrast, BAX and BAK proteins play important roles in the normal morphogenesis of the mitochondria by activating assembly of the fusion GTPase, Mfn2, thereby promoting fusion of the mitochondria [35]. However, we did not find that loss of BAX and BAK altered mitochondrial morphology or dynamics under normal cell culture conditions (Movie S1) compared with wild-type cells (Movie S2). Bid BH3 peptide altered mitochondrial morphology and dynamics (Movies S3) compared to mutant BH3 peptide (Movie S3 and Movie S4). Interestingly, we could not rescue mitochondrial fission by a dominant negative Drp1 or mdivi-1, a pharmacological inhibitor of mitochondrial fission. Since the process of mitochondrial fission is not fully understood [14], we cannot exclude the possibility that unidentified regulator(s) of fission might be responsible for cell death in the absence of BAX/BAK in the presence of BH3 peptides. Much of the previous work has primarily focused on the regulation of mitochondrial fission by BAX and BAK and not by the pro-survival BCL-2 proteins. A recent study did demonstrate that the pro-survival protein BCL-X_L_ binds to DRP1 which leads to an increase in the GTPase activity of DRP1 [27]. We also observed that DRP1 coimmunoprecipitates with BCL-X_L_. Moreover, this interaction between the two proteins was increased when BH3 peptide was added to the cells. Our current findings, however, does not distinguish whether the BH3 peptide binding to pro-survival proteins is permissive for mitochondrial fission to occur, or whether the BH3 binding to pro-survival protein directly promotes mitochondrial fission.

The physiological implication of our findings presently remains unknown. The BAX and BAK deficient animals are not embryonic lethal and do not display severe developmental defects [36] when compared to BCL-XL, MCl-1 or caspase-9, deficient animals which all display severe developmental defects [37]–[39]. The mechanism by which cells undergo developmental cell death in the absence of BAX and BAK remains unknown. But it suggests that there are other death mechanisms initiated during development in the absence of BAX and BAK to obtain viable mice. We speculate that BH3 proteins engage prosurvival BCL-2 proteins to trigger mitochondrial fission and cell death in the absence of BAX and BAK during development. The mitochondrial fission results in dysfunctional mitochondria which could trigger cell death in the absence of BAX/BAK. In summary our data provide evidence that pro-survival proteins can regulate mitochondrial fission and death in the absence of BAX and BAK.

## Methods

### Cell lines

Wild type and *Bax^−/−^/Bak^−/−^* mouse embryonic fibroblasts were kindly provided by Dr. Craig Thompson. Wild type and *Bax^−/−^/Bak^−/−^* Baby mouse kidney epithelial cells were kindly provided by Dr. Eileen White. Cells were cultured in Dulbecco's modified essential media (DMEM), supplemented with 10% heat-inactivated Fetal Bovine Serum (FBS), 100 U/ml penicillin, 100 µg/ml streptomycin and 20 mM Hepes. All cell culture reagents were purchased from GIBCO.

### Peptides

Peptides containing 8 D-arginine were synthesized by Tufts University Core Facility and purified by HPLC. The N-terminus and C-terminus of the peptides were blocked by an acetyl and amide group respectively. Peptide sequences are the following:

BID BH3: EDIIRNIARHLAQVGDSMDR,
Mutant BID BH3: EDIIRNIARHAAQVGASMDR,
BIM BH3: MRPEIWIAQELRRIGDEFNA,
BAD BH3: LWAAQRYGRELRRMSDEFEGSFKGL,
Mutant BAD BH3: LWAAQRYGREARRMSAEFEGSFKGL,
NOXA A BH3: AELPPEFAAQLRKIGDKVYC,
NOXA B BH3: PADLKDECAQLRRIGDKVNL

### Measurement of cell death

Cell death was assessed by the release of lactate dehydrogenase (LDH) into the surrounding medium using a cytotoxicity detection kit from Roche Applied Science. Percentage of cell death was calculated by the amount of LDH released in the medium, divided by the total LDH released after treatment of cells with 1% Triton X-100. Flow cytometry was also used to detect PI positive cells stained according to manufacture's protocol (BD Biosciences).

### Immunoblot Analysis

Protein expression was analyzed in total cell by lysing cells with 1× cell lysis buffer (Cell Signaling) supplemented with 1 mM phenylmethylsulfonyl fluoride. Protein concentration was determined using the Bio-Rad protein assay. 50 µg of total cell lysate were resolved on a 10% or 12% sodium dodecyl sulfate-polyacrylamide gel (Bio-Rad) and transferred to a Hybond-ECL nitrocellulose membrane (Amersham). Membranes were blocked in 5% milk in Tris-buffered saline-Tween 20 buffer. Primary antibodies used were Bok antibody (Cell signaling), Bcl-X_L_ antibody (Santa cruz), DRP1 antibody (H-300, sc 32898), Flag antibody (Sigma), Cytochrome c antibody (Mitosciences), COX-1 antibody (BD Pharmingen) and alpha-tubulin antibody (Sigma clone B-5-1-2) at 1∶2,000. Secondary antibodies used were horseradish peroxidase-linked anti-mouse or anti-rabbit IgG (Cell Signaling) 1∶1000. SuperSignal chemiluminescent substrate (Pierce) was used to develop the blot. A representative blot is shown above of three independent experiments.

### Live cell imaging

Cytochrome c release was monitored using cytochrome c tagged with GFP. Mitochondrial membrane depolarization was assessed by TMRE release. Mitochondria morphology was determined by Mitotracker CMX-ROS (Invitrogen) counterstain. Dynamic live cell imaging was performed on a Yokogawa spinning disc confocal fitted on a Nikon TE2000U microscope enclosed in 37C heated CO_2_ chamber, housed at the Northwestern University Cell Imaging Facility. Image acquisition was performed by Hamamatsu 9100C electron-multiplication CCD camera through a 100× objective lens (N.A. 1.46). Care was taken during image acquisition to ensure that there were no saturated pixels. Image analysis was performed by MetaMorph (version 6.3r5) software. The release of TMRE upon membrane depolarization caused a decrease in intramitochondrial fluorescent intensity. To track the time-dependent TMRE fluorescent intensity changes, a region was drawn along the cell outline. This region was then transposed to a cell-free region in the same field of view adjacent to the cell being imaged. To obtain the mitochondrial/diffuse index, the intensity standard deviation [40] within these two regions was ratiometrically compared over time as: (I_Cell_ − I_Background_)/I_Background_; wherein I_Cell_ represents the intensity standard deviation of within the region outlining the cell, and I_Background_ represents the intensity standard deviation within the exact same region transposed to a cell-free area. The mitochondria/diffuse index thus allowed us to simultaneously correct for background fluorescence fluctuations and track TMRE release.

### Mitochondrial Transition Pore Assay

To assess for the mitochondrial transition pore opening, *Bax^−/−^/Bak^−/−^* MEFs were treated with corresponding BH3 peptides for 1 hour, trypsinized and labeled for flow cytometry using MitoProbe™ Transition Pore Assay Kit (Invitrogen) according to manufacture's protocol. The change in mean Calcein fluorescence of the mitochondria before and after addition of ionomycin indicates activation of the mitochondrial permeability transition pore.

### Mitochondria isolation and cytochrome c release

Wild type or *Bax^−/−^/Bak^−/−^* MEFs were collected in mitochondrial isolation buffer (250 mM sucrose, 10 mM Tris-Hcl pH 7.4, 0.1 mM EGTA. Mitochondria were obtained by mechanical cell disruption using a dounce homogenizer followed by 10 expulsions through a 27 gauge syringe and differential centrifugation. Isolated mitochondria were then re-suspended in experimental buffer (125 mM KCl, 10 mM Tris-MOPs, 5 mM glutamate, 2.5 mM malate, 1 mM KPO, 10 uM EGTA-Tris), and incubated with appropriate peptides for 30 minutes at room temperature. Cytochrome c release was assessed by ELISA using a mouse cytochrome c immunoassay from R& D Systems. As a control to assess for total (100%) cytochrome c release, isolated mitochondria were treated with 0.5% Tx-100 (Sigma) while the minimum value is cytochrome c release from cells treated with DMSO. Percentage cytochrome c release was calculated according to the following equation: %cyto c = (X-min cyto c)/(Total cyto c- min cyto c), whereby X is the value obtain for each experimental condition.

### Retroviral transfection

Packaging cell line PLAT-E (kind gift of T. Kitamura) were transfected using Mirus *Trans*IT Transfection reagent (Mirus Bio Corporation) according to the manufacturer's protocol. 48 hours post transfection, medium containing virus was supplemented with 8 µg/ml polybrene (Sigma) for cell line infection and applied to MEFs. The pSiren vector (Clontech) was used to express short hairpin RNA (shRNA) sequences for Bok (5′-CAGATCCGTCCCAGCGTAT-3′) and *Drosophila melanogaster* HIF (dHIF) (5′-GCCTACATCCCGATCGATGATG-3′). Cytochrome c GFP construct was a kind gift from Dr. D.R. Green. DN-Drp-1 construct was a kind gift from Dr. R. Youle, which we re-cloned into pBabe GFP. Infected cells were selected with corresponding selection markers, and for GFP expressing cells, selection was achieved by sorting using the DakoCytomation MoFlo high speed multilaser droplet cell sorter at 488 nm.

### Electron Microscopy

Treated cells were rinsed with PBS and fixed for 1 hour in 2.5% glutaraldehyde at room temperature followed by another rinse in 0.1 M cacodylate buffer. Coverslips were then incubated in secondary fixative of 2% osmium tetroxide for 30 minutes. Samples were then en-bloc stained with 3% uranyl acetate. Fixed cells were dehydrated in a graded series of ethanol and embedded in araldite and epon mixture. Following sectioning, the samples were contrasted with 6% uranyl acetate and Reynols lead. Images were taken on the JOEL 1220 Transmission Electron Microscope. Random cells were chosen for imaging and aspect ratio of all mitochondria within a cell section were calculated using the MetaMorph software (version 6.3r5).

### Immunoprecipitation

Bcl-x_L_ was immunoprecipitated using the FLAG® Tagged Protein Immunoprecipitation Kit (Sigma) per manufacturer's protocol. Briefly, *Bax^−/−^/Bak^−/−^* MEFs overexpressing FLAG- Bcl-x_L_ were plated in 10 cm plates. At 50% confluency, cells were treated with 100 µM of BID or mutant BID BH3 peptide for 10 minutes. Cells were washed in PBS and Cell Lysis Buffer (50 mM Tris HCl, pH 7.4, with 150 mM NaCl, 1 mM EDTA, and 1% TRITON™ X-100) was added. Total protein was determined by Bradford Assay. Flag tagged Bcl-X_L_ were immunoprecipiated with ANTI-FLAG M2 affinity gel. Equal protein was added to the gel and incubated overnight on a roller shaker at 4°C. Immunoprecipitates were separated on 12% poly-acrilimide gels and detected using anti-DRP1 antibody (H-300, Santa Cruz Biotechnology, 1∶200) and Flag antibody (Sigma) as loading control.

### Microinjection

*Bax^−/−^/Bak^−/−^* MEFs were microinjected with BID or mutant BID peptide that does not contain 8D-arginine sequence. The peptides were dissolved in 10 mM Tris and Dextran FITC was added as a marker for identification of injected cells. Microinjection was carried out on an Axovert 135 microscope equipped with an Ependorf Femtojet microinjector. Cells were injected using Femtotip II injection capillary at a pressure of 22 hPa. Injected cells were viewed 30 minutes following injection using a fluorescence microscope LSM510 and pictures were captured using a 63× lens objective.

### Other reagents

MDIVI compound was purchased from Ryan Scientific and was resuspended in DMSO. 3-Methyladenine, staurosporine, and cyclosporin A were purchased from Sigma Aldrich. Q-VD-OPh was purchased from R&D Scientific. Mitotracker CMX-ROS and tetramethylrhodamine ethyl ester (TMRE) were purchased from Invitrogen.

## Supporting Information

Figure S1 FITC tagged peptide uptake in Bax−/−/Bak−/− MEFs. (A) Bax−/−/Bak−/− MEFs treated with DMSO, (B) 100 µM mutant BID or (C) 100 µM BID BH3 peptides that were tagged to FITC for 5 minutes. Flow cytometry was performed to assess for GFP positive cells.
(0.53 MB TIF)

Figure S2 Bax−/−/Bak−/− MEFs overexpressing BCL-XL are not protected against peptide induce cell death. (A) LDH release of Bax−/−/Bak−/− MEFs overexpressing empty construct or BCL-XL when treated with increasing concentration of BID or mutant BID BH3 peptide at 24 hours. Mean values±SEMs of 3 independent experiments are shown. (B) Western Blot analysis of whole cell lysate of Bax−/−/Bak−/− MEFs, overexpressing empty construct or BCL-XL.
(0.69 MB TIF)

Figure S3 CsA inhibits the permeability transition pore of Bax−/−/Bak−/− MEFs. Bax−/−/Bak−/− MEFs were loaded with Calcein AM in the presence of cobolt choride for mitochondria labeling. Following mitochondrial Calcein uptake, only cells that were treated with CsA allowed for Calcein retention following treatment with ionomycin.
(0.50 MB TIF)

Movie S1 Bax/Bak Null MEFs Mitotracker Red
(0.85 MB MPG)

Movie S2 Wild-type MEFs Mitotracker Red
(1.75 MB MPG)

Movie S3 Bax/Bak Null MEFs+Bid BH3peptide Mitotracker Red
(0.63 MB MPG)

Movie S4 Bax/Bak null MEFs+mutant BH3 peptide Mitotracker Red
(0.50 MB MPG)
