# Supplementary figures and images for: BH3 Peptides Induce Mitochondrial Fission and Cell Death Independent of BAX/BAK
Source: PLoS One. 2009 May 21;4(5):e5646. doi: 10.1371/journal.pone.0005646 (PMC2681411; doi:10.1371/journal.pone.0005646)

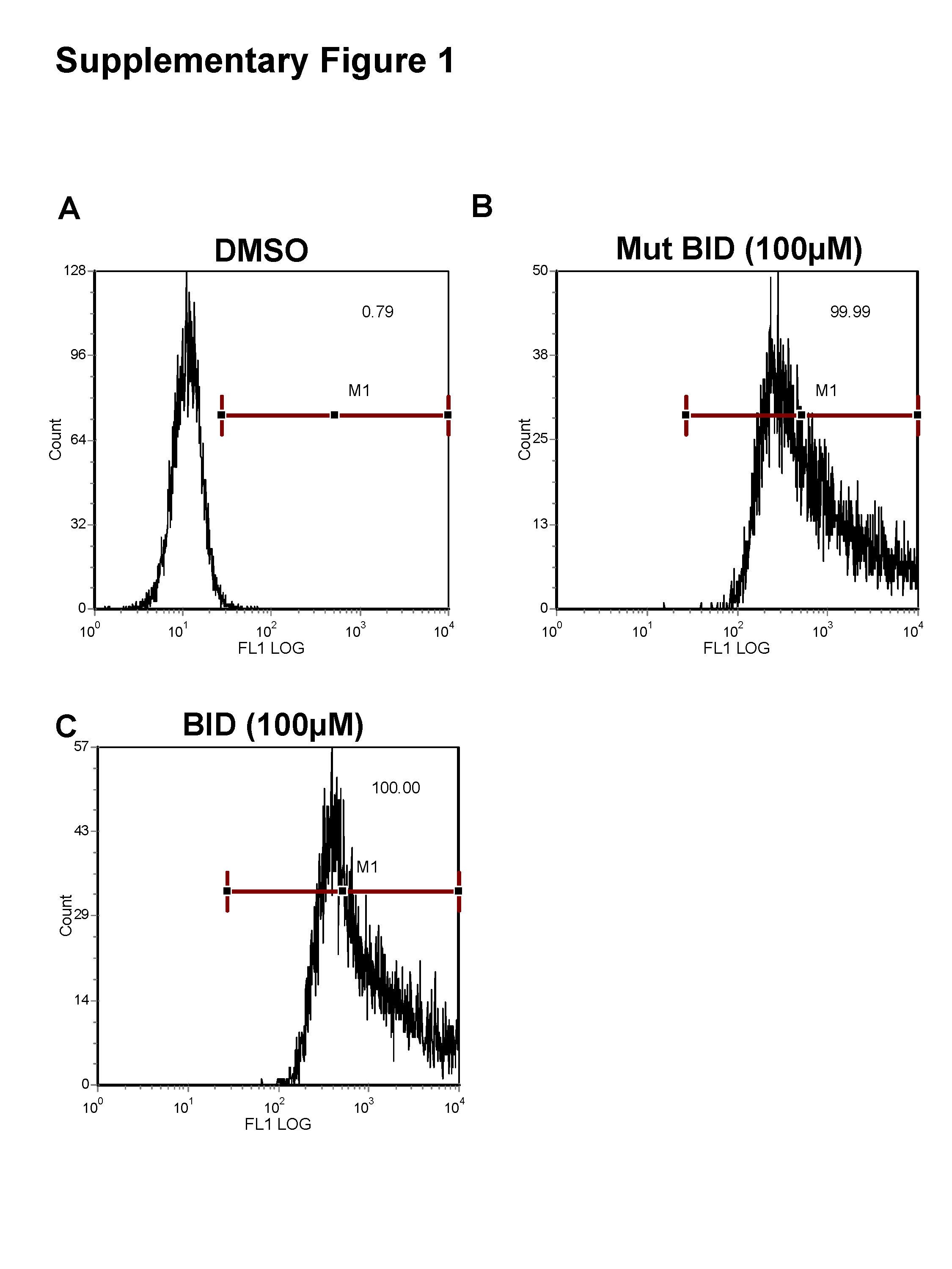

Supplement: Figure S1 — FITC tagged peptide uptake in Bax−/−/Bak−/− MEFs. (A) Bax−/−/Bak−/− MEFs treated with DMSO, (B) 100 µM mutant BID or (C) 100 µM BID BH3 peptides that were tagged to FITC for 5 minutes. Flow cytometry was performed to assess for GFP positive cells. (0.53 MB TIF) [file pone.0005646.s001.tif]

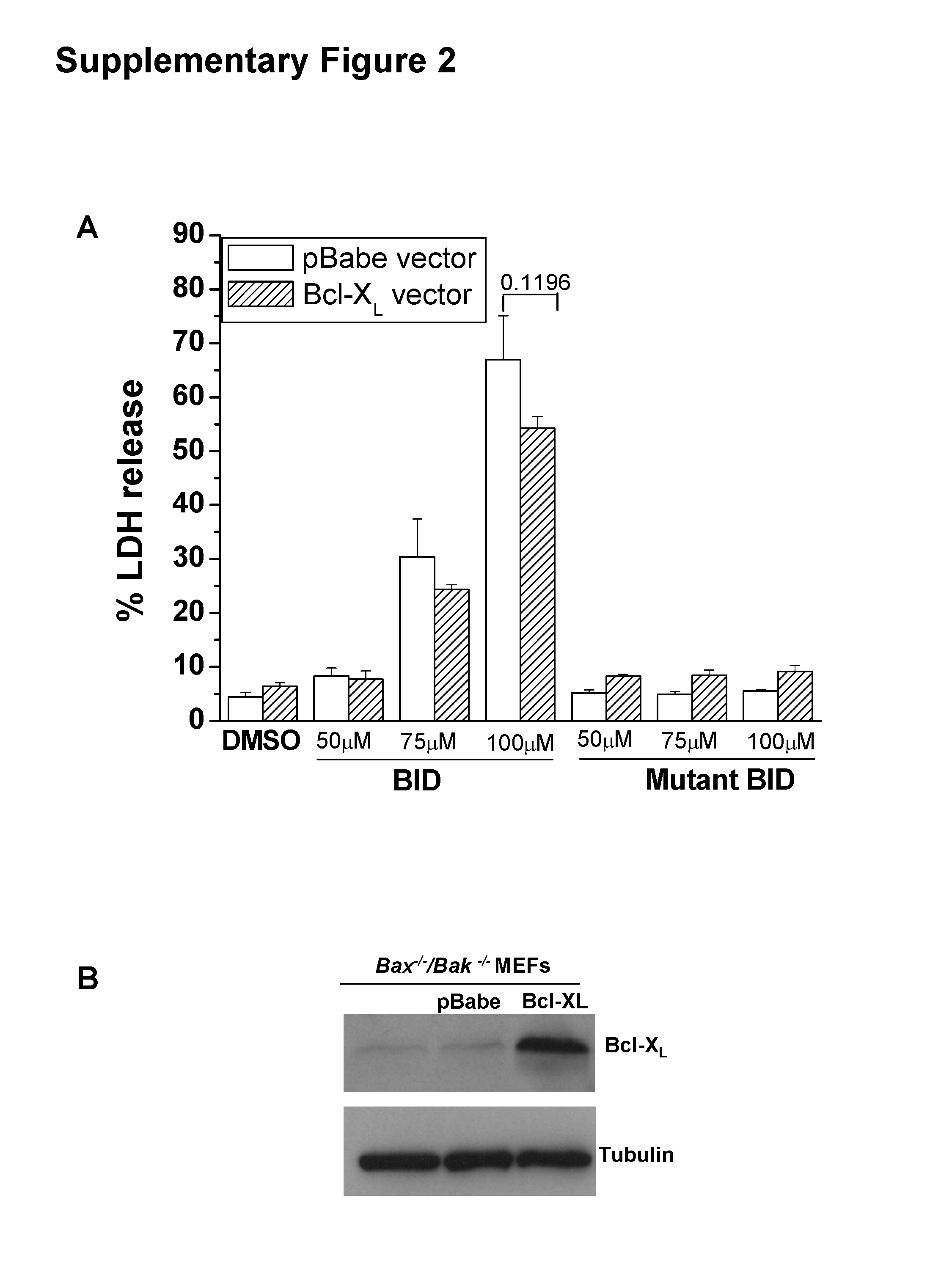

Supplement: Figure S2 — Bax−/−/Bak−/− MEFs overexpressing BCL-XL are not protected against peptide induce cell death. (A) LDH release of Bax−/−/Bak−/− MEFs overexpressing empty construct or BCL-XL when treated with increasing concentration of BID or mutant BID BH3 peptide at 24 hours. Mean values±SEMs of 3 independent experiments are shown. (B) Western Blot analysis of whole cell lysate of Bax−/−/Bak−/− MEFs, overexpressing empty construct or BCL-XL. (0.69 MB TIF) [file pone.0005646.s002.tif]

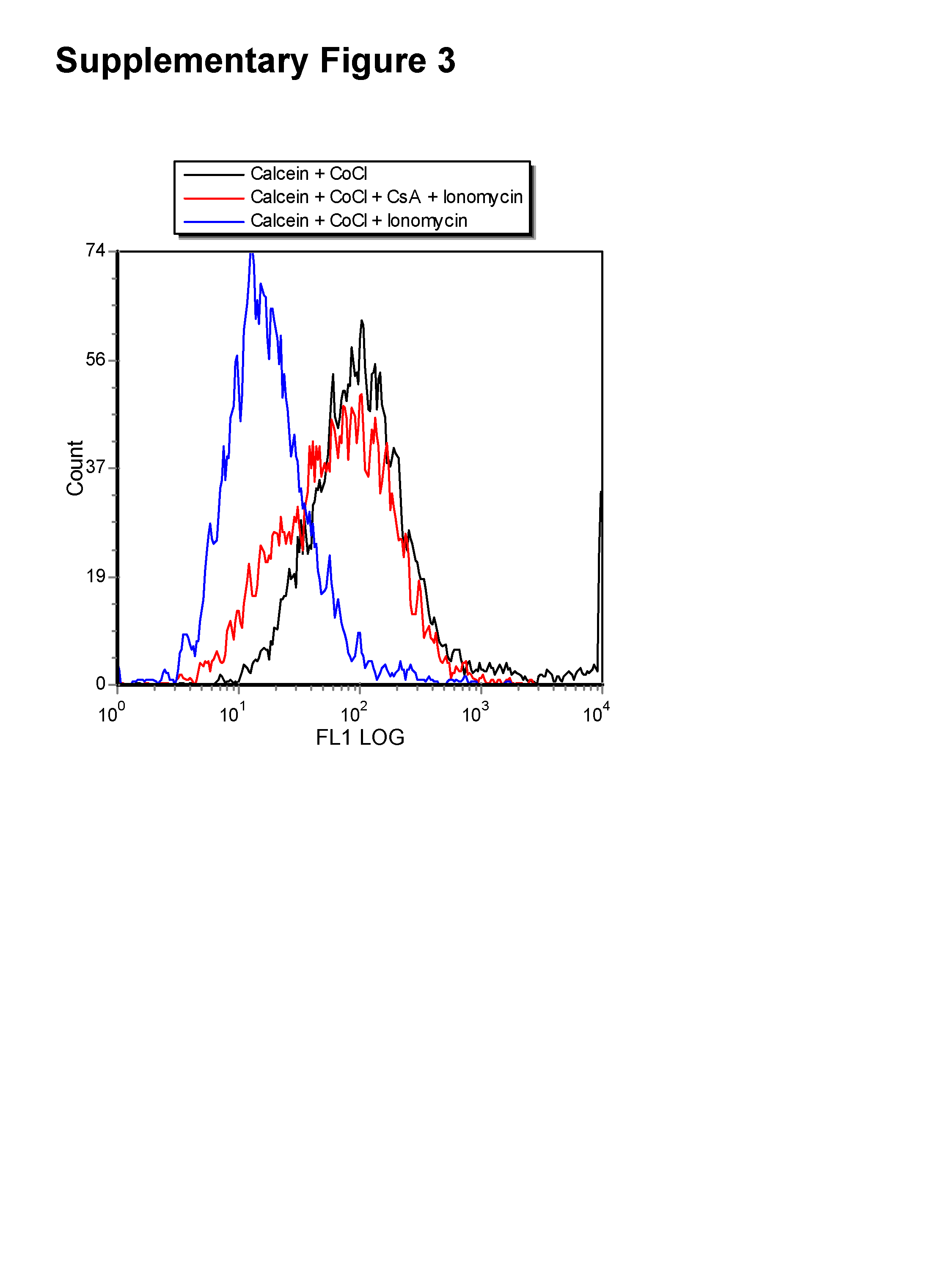

Supplement: Figure S3 — CsA inhibits the permeability transition pore of Bax−/−/Bak−/− MEFs. Bax−/−/Bak−/− MEFs were loaded with Calcein AM in the presence of cobolt choride for mitochondria labeling. Following mitochondrial Calcein uptake, only cells that were treated with CsA allowed for Calcein retention following treatment with ionomycin. (0.50 MB TIF) [file pone.0005646.s003.tif]
